# Assessment of occupational exposure to lead among workers engaged in a city bus garage in Addis Ababa, Ethiopia: a comparative cross-sectional study

**DOI:** 10.1186/s12995-024-00422-9

**Published:** 2024-06-20

**Authors:** Merihatsidik Tesema Abebe, Abera Kumie, Samson Wakuma Ayana, Teshome Assefa, Wossenyeleh Ambaw

**Affiliations:** 1 Department of Public Health Emergency Management Center, Ethiopian Public Health Institute, Addis Ababa, Ethiopia; 2 Department of Preventive Medicine, School of Public Health, College of Health Sciences, Addis Ababa University, Addis Ababa, Ethiopia; 3 Department of Health Nutrition, Ethiopian Public Health Institute, Addis Ababa, Ethiopia; 4 Department of Food Science, Ethiopian Food and Drug Authority, Addis Ababa, Ethiopia

**Keywords:** Blood lead levels, Exposure factors, Garage workers, Lead exposure, Occupational exposure

## Abstract

**Background:**

Lead is one of the most nonessential toxic heavy metal agents found in automotive garages. The occupational exposure of garage workers to lead commonly poses acute and chronic health risks that can be prevented. In Ethiopia, there have been limited studies on lead exposure among garage workers, who overemphasize exposure to lead. This study aimed to assess occupational blood lead levels and associated factors in garage workers using a cross-sectional comparative design.

**Methods:**

A comparative cross-sectional study design was used to compare the occupational blood lead levels of 36 randomly selected garage workers and 34 office workers who were matched by age and sex. Blood specimens were collected by trained medical laboratory experts. The collected blood samples were tested in a certified laboratory using a microwave plasma atomic emission spectrometry (MP-AES) device at a wavelength of 405.78 nm. Excel and SPSS Version 26 were used for data management and analysis, respectively.

**Results:**

The mean (SD) age of the exposed group was 39.0 (7.5) years, whereas the mean age of the unexposed group was 38.0 (6.1) years. The occupational mean (SD) blood-lead-level in the exposed groups was 29.7 (12.2) µg/dl, compared to 14.8 (9.9) µg/dl among the unexposed groups. The mean blood-lead level among the exposed workers was significantly different from that among the unexposed workers (*P* < 0.01). Of all the study participants, only 22.2% of the exposed groups had blood lead levels higher than the World Health Organization’s recommended limit of 40 µg/dl. The main significant predictors of occupational blood-lead-level exposure among workers were extra working hours, service years, and having a previous (prior) employment history in a garage. The occupations of the two groups did not significantly differ in terms of blood-lead levels (*p* > 0.05).

**Conclusions:**

The BLL of the Garage workers was significantly greater than that of the Non-Garage workers. Hence, it is advised that garage management should encourage workers to use exposure prevention methods, such as washing their hands before eating and taking showers after the completion of work, by providing regular occupational safety training.

## Introduction

Around the world, from small to large garage sites, most garage workers are occupationally exposed to heavy metals. One of the most nonessential and harmful types of heavy metals for public health concerns that is still found in occupational work and the environment is lead. Lead is commonly observed as an occupational hazard for workers who are working in painting, welding, battery maintenance, mechanics, and electricity [1, 2]. Garage workers are more exposed to lead than non-garage workers. However, millions of people are exposed to lead through inhalation, ingestion, and skin contact pathways that occur in workplaces and in the environment [1, 3]. Exposure to lead particles poses significant health problems for workers and affects the reproductive health system, leading to conditions such as memory impairment, kidney failure, intestinal and lung cancer, central nervous system disorders, anaemia, hypertension, and other conditions [4, 5]. The 2019 Institute for Health Metrics and Evaluation (IHME) estimation report indicated that lead exposure caused 900,000 deaths annually and 21.7 million years of loss of health (also known as DALYs) due to its long-term impact on health [6, 7]. Given this burden, limited effective occupational health treatments exist for 20–50% of workers in industrialized countries and 5–10% of workers in developing countries [8]. However, more people die each year from occupational diseases and injuries than from malaria, with nearly 2 million deaths worldwide each year. Most workers are susceptible to lead exposure due to environmental and occupational exposure, in addition to a lack of safety training and awareness, the inability to use personal protective equipment (PPE), and spending more time at work [9]. Hence, to prevent inhalation or ingestion of occupational lead among garage workers, PPE, hygiene and sanitation practices, ventilation, and regular checkups are necessary and available to reduce the transmission of occupational lead dust, fumes, and other particulate matter in the workplace.

Studies around the world indicated that automotive workers in developed countries checked their blood lead levels regularly, while garage workers who were working in developing countries, including Ethiopia, did not test their blood lead levels due to ineffective regular provisions to monitor the exposure regularly [10, 11]. It was also observed that the garage workers did not use PPE or safety measures while they were working. The probability of garage workers being exposed to lead is greater than that of non-garage workers [1, 5, 12]. According to a previous study conducted in Khyber, Pakistan, the mean measured blood lead level in exposed groups was 65.3 ± 41.9 µg/dl, whereas in the non-exposed groups, it was 21.7 ± 17.6 µg/dl [1]. Consequently, the BLL is a biomarker of blood, hair, urine, teeth, or other body parts [1, 5, 13, 14].

In Addis Ababa, Ethiopia, there is only one large Anbessa city bus service enterprise that provides transport services in and around the city of Addis Ababa. Four bus garage sites are located in four different sub-cities of Addis Ababa. Occupational lead exposure studies have been conducted among garage workers at these sites. Some studies on the occupational lead exposure of automotive workers conducted in Jimma and Harer, Ethiopia, did not include workers working at Anbessa city bus service enterprise (ACBSE) garage sites [5, 12]. In Jimma town, the mean blood lead level (BLL) of automotive garage workers was 19.75 ± 4.46 µg/dl, which was significantly greater than that of non-exposed workers (mean blood lead level 10.73 ± 2.22 µg/dl). However, the study did not divide the garage workers into groups of workers working mechanistically, painters, welders, or electricians. A study conducted in Harar, Ethiopia, in 2018 measured the blood pressure and hematological parameters of the garage workers, which did not indicate the exposure of lead levels in the blood. In another study that was conducted in Addis Ababa, Ethiopia, only urinary δ-ALA levels and not lead exposure levels were measured in the blood of lead acid battery repair workers [15]. Since exposure lead levels in the blood have a half-life of 40 to 120 days but remain in bones for a long time, constituting, on average, 95% of the body’s total lead burden, measuring blood lead levels is typically a valid indicator of lead exposure in the workplace [16].

Thus, the purpose of this study was to assess the exposure of blood lead levels and their associated factors in Anbessa city bus service (ACBSE) garage workers in Addis Ababa, Ethiopia, by comparing them with non-exposed groups from non-garage sites.

## Materials and methods

### Study area and period

**Fig. 1:**
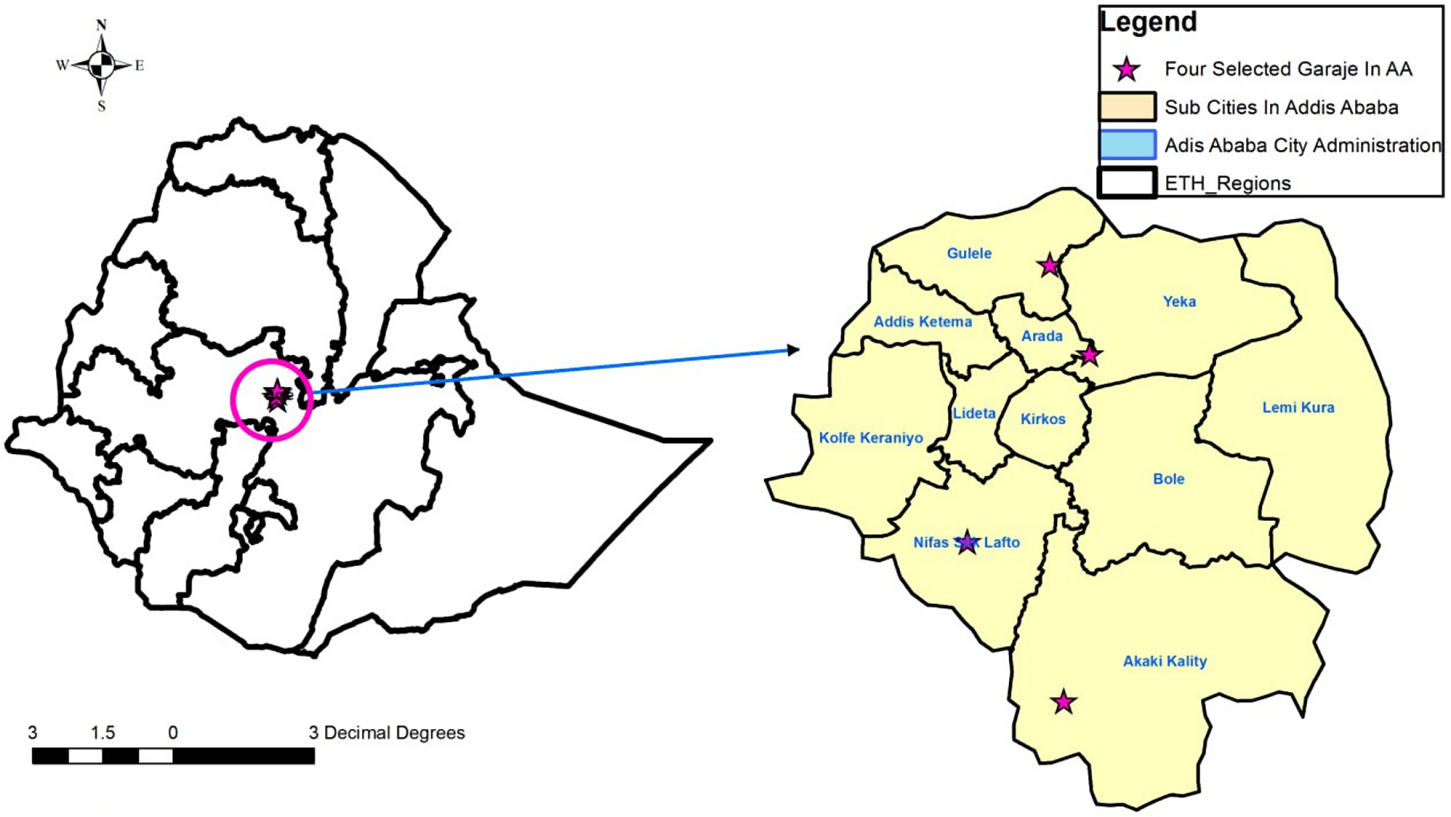
Study area of the anbessa bus city service enterprise garage sites in four sub-cities of Addis Ababa, Ethiopia

The study was carried out in Addis Ababa, Ethiopia, among garage workers at four Anbessa City Bus Service Enterprise (ACBSE) garage sites by comparing them to non-garage workers from March to September 2023. The Enterprise is one of the oldest and largest modern public services in the city. It has approximately 590 garage workers. The Enterprise was established 80 years ago, during Emperor Haile Selassie’s reign. Currently, it has four standard garage sites at Yeka, Gulelle, Nefas Silk, and Akaki-Kality Sub-cities of Addis Ababa (Fig. 1).

### Study design and study subjects

A comparative cross-sectional study of Anbessa City bus service enterprise garage workers and non-garage workers who are working at the Ethiopia Public Health Institute (EPHI) as office workers was conducted. Blood samples were taken from 36 occupationally exposed garage workers (31 males and 5 females) and 34 occupationally unexposed office workers (28 males and 6 females) for blood lead level analysis.

### Inclusion and exclusion criteria

Garage workers involved in one of the following job positions—mechanics, electricians, welders, or painters—had at least one year of work experience in garage work. Workers with a previous history of chronic cases, such as diabetes or hypertension, and those who were following their health status in hospitals and rotating from one job position to another were excluded. The unexposed groups were randomly selected as a comparison group if they did not have any previously intended occupational lead exposure history and had approximately matched demographic characteristics, particularly age and sex, as exposed groups.

### Sample size

The sample size was calculated using a double-mean-standard deviation comparison formula.

$$Sample\;size\;(n)\; = \;\frac{{(\sigma _1^2 + \sigma _2^2)({Z_\beta } + {Z_{\alpha /z}})2}}{{(d)2}}$$

According to a previous study, the mean ± SD was 0.42 ± 0.13 µmol/L in the exposed groups, and the mean ± SD was 0.32 ± 0.07 µmol/L in the unexposed groups [17]. The open Epi version 3 software calculator was used to estimate the sample size with a specified significance level (0.05) and power (95%) for both the exposed and non-exposed groups. In the first calculation, we obtained n_1_ = 29 and n_2_ = 29 sample sizes. By adding a 15% nonresponse rate, the calculated sample sizes were n_1_ = 34 and n_2_ = 34. However, using the approximate proportional allocation technique of the 34 calculated sample sizes to the total population size, 36 garage workers were occupationally exposed for this study.

### Sampling

#### Selection of exposed groups

Before sampling the participants at the garage sites, a preliminary assessment was conducted to check the functionality of the Anbessa City Bus Service Enterprise garage sites and the availability of workers. At that time, 590 occupational garage workers were working at four sites in the Anbessa City Bus Service Enterprise (ACBSE). Of these 590 garage workers, only 530 fulfilled the inclusion criteria and were used for the sampling frame. However, the proportional allocation method was conducted based on the 34 calculated sample sizes for each occupational job position (mechanics, electricians, painters, and welders) of the garage workers, and 36 sampled garage workers were selected randomly (Table 1).

#### Selection of unexposed groups

Unexposed groups were selected from among office workers who were working at the Ethiopian Public Health Institute in Addis Ababa, Ethiopia, for comparison purposes. The comparison group was considered to not have a history of chronic cases of lead exposure and was matched for age and sex as garage workers. According to the Institute’s human resources record, some of the office workers were working as supportive staff, and others were health professionals. From these lists, those who fulfilled the inclusion criteria were identified as the unexposed group. The EPHI has a total of 500 health professionals and 300 supportive staff members. However, of all office workers, only 300 health professionals and 200 supportive staff members fulfilled the inclusion criteria and were used for the sampling frame. With a proportional allocation technique of the 34 calculated sample sizes for each occupational job position, 34 unexposed groups were randomly selected for this study (Table 1).

**Table 1:** Sample size proportional allocation based on occupational job position of garage and non-garage workers in four garage sites of ACBSE and EPHI in Addis Ababa, Ethiopia, 2023

No.	Major garage sites	Number of garage workers	Sampled workers
1	Gerji	164	10.5 (11)
Job position	Mechanics	87	5.6 (6)
	Electrician	20	1.3 (1)
	Welders	49	3.1 (3)
	Painters	8	0.5 (1)
2	Jemo/Mekanissa	117	7.5 (8)
Job position	Mechanics	46	2.9 (3)
	Electrician	15	1.0 (1)
	Welders	42	2.9 (3)
	Painters	14	0.9 (1)
3	Kality	148	9.5 (10)
Job position	Mechanics	48	3.1 (3)
	Electrician	46	2.9 (3)
	Welders	28	1.8 (2)
	Painters	26	1.7 (2)
4	Shegolle	101	6.5 (7)
Job position	Mechanics	45	2.9 (3)
	Electrician	26	1.7 (2)
	Welders	30	1.9 (2)
	Painters	0	0
	Overall	530	36
5	EPHI (non-garage site)	500	34
Job position	Health Professionals	320	21.8 (22)
	Supportive Staffs	180	12.2 (12)

### Reagents and laboratory glassware

Five series of analytical standard solutions of lead were prepared—20 (µg/dl), 40 (µg/dl), 60 (µg/dl), 80 (µg/dl), and 100 (µg/dl)—by serially diluting a 1000 mg/L commercial stock calibration standard solution of lead. All chemicals and reagents used in the laboratory were of analytical grade.

### Blood sample collection

Four-milliliter venous blood samples were collected from each of the 36 occupationally exposed workers and from each of the 34 occupationally unexposed workers using Pb-free, separate labelled vacationer tubes containing 7.2 mg of K2EDTA by trained medical laboratory professionals. To reduce the contamination of samples, the enterprise clinics and other safety materials were used as blood collection sites. The properly collected samples were transported to the EPHI laboratory using a cold box, stored at 4 °C and preserved at -20 °C until the digestion time of analysis.

### Sample preparation

After accurate measurement, a 2 ml portion of each whole-blood sample was transferred to a digestion beaker, and 10 ml of a freshly prepared mixture of concentrated nitric acid and hydrogen peroxide (HNO_3_ (70%) and H_2_O_2_ (30%) (6:4 v/v)) was added, and the mixture was allowed to stand for 10 min. The beakers were covered with a watch glass and then heated at 110 °C for 1–2 h. The digests were again treated with a few mixtures of HNO_3_ and H_2_O_2_ while increasing the hotplate temperature to approximately 250 °C until digestion or a clear solution was obtained. The excess acid mixture was evaporated until the clear solution remained approximately semidry. Then, we cooled and filtered each clear solution and transferred it to a volumetric flask (100 ml) by diluting it to the mark with deionized water. At the same time, blanks (without samples) were prepared in triplicate using deionized water. Finally, each prepared clear solution was stored and refrigerated at -4 °C until laboratory analysis. The procedures for sample preparation were based on the National Institute of Standards and Technology (NIOSH) fourth edition Manual for Analytical Methods (NMAM) on Method 8005 Issue 2 (1994) and validated published literature [1, 18, 19].

### Blood lead level analysis

After the instrument parameters were optimized, the lead levels in the blood of the study subjects were measured by microwave plasma atomic emission spectroscopy (MP-AES) with an Agilent Model 4210 spectrophotometer at 405.781 nm. The low detection limit (BDL) of the instrument for reading the sample was at or above the 0.1 µg/dl detection limit. At this level, the instrument cannot read the sample. The calibration curve of the instrument was generated by running five series of standard solutions of lead. Triplicate samples were analysed, and the average results for each measured sample were taken. A recovery test was performed on four prepared blood samples by selecting randomly from the spiked and unspiked samples with a known sample solution. Then, 99.6% of the average percentage recovery was obtained within the recovery range (80–110%) of the samples [1, 20]. In addition to the EPHI-accredited laboratory, another cross-checking Ethiopian Food and Drug Authority (FDA) laboratory was used to check the accuracy and precision of each sample result.

### Data collection on exposure factors

In addition to measuring the occupational blood lead level exposures of the study participants, data on socio-demographic factors, behavioural factors, and occupational factors of respondents were collected using an interviewer-administered pretested questionnaire. The questionnaire was designed to provide information on factors associated with the measured occupational blood lead levels by preparing it in English and translating it to a local language for those who do not understand English. The questionnaire was back-translated to English for consistency checking.

### Statistical processing and analysis of data

All the data were cleaned and managed in Excel and analysed using the Statistical Package for Social Science (IBM SPSS, Chicago, America) (SPSS version 26) software. Descriptive statistics were used to display the demographic, behavioral, and occupational characteristics in the form of frequencies, means, and percentages. An independent t test was used to compare the statistically significant differences between the exposed and unexposed groups in terms of occupational exposure to blood lead. One-way ANOVA was used to investigate the variation in blood lead levels with specific occupational job positions (mechanics, electricians, welders, and painters) of the study participants, such as workers.

Variables were selected for multiple linear regressions by first conducting a simple linear regression of the dependent variable (BLL) with each independent variable (service years, age, extra working hours, and other independent variables). Then, we selected only variables that had a p value less than 0.2 for multiple linear regression analysis. Multiple linear regression analysis was used to determine the effect of independent factors on the occupational blood lead level of the study participants by considering a p value < 0.05 to indicate statistical significance. However, before applying the regression method, all assumptions for linear and multiple regressions were checked. Diagnostics for multicollinearity among the independent variables were performed using the variance inflation factor. Independent variables with variance inflation factor values > 10 were considered indicative of multicollinearity and removed from the multiple linear regressions.

## Results

### Socio-demographic characteristics of the study participants

A total of 70 respondents (36 garage workers and 34 office workers) were available for the data analysis. The socio-demographic characteristics of the respondents are presented in Table 2. The mean age of the exposed group was 39.0 ± 7.5 years, ranging from 27 to 50 years, compared with that of the unexposed group; the mean age was 38.0 ± 6.1 years, with a range of 28 to 49 years. Among the study participants, 86.1% and 82.4% were males in the exposed and unexposed groups, respectively. There were more (80.6% and 79.5%) married than single (19.4% and 23.5%) individuals in the exposed and unexposed groups, respectively. The majority (58.3%) of the exposed groups had diplomas compared to the majority (85.3%) of the unexposed groups who had degrees and above. According to Ethiopian Birr, the majority (72.2%) of the exposed group and 91.2% of the unexposed group were paid a monthly income above 6,000 .

**Table 2:** Socio-demographic and economic characteristics of the exposed groups (*n* = 36) and unexposed groups (*n* = 34) in Addis Ababa, Ethiopia, 2023

Variables	Exposed group *n* (%)	Unexposed group *n* (%)
Age (yrs.)		
18–30	8 (22.2)	6 (17.7)
31–44	16 (44.4)	22 (64.7)
>=45 **Mean ± SD**	12 (33.3) **39.0 ± 7.5**	6 (17.7) **38.0 ± 6.1**
Sex:		
Male	31 (86.1)	28 (82.4)
Female	5 (13.9)	6 (17.6)
Education:		
Below diploma	7 (19.4)	4 (11.8)
Diploma	21 (58.3)	1 (2.94)
Degree and above	8 (22.2)	29 (85.29)
Marital status		
Single	7 (19.4)	8 (23.5)
Married	29 (80.6)	26 (76.5)
Monthly income:		
<=6,000	10 (27.8)	3 (8.8)
> 6,000	26 (72.2)	31 (91.2)

### Behavioural characteristics of exposed groups

Regarding PPE users, 17 (47.2%) participants in the exposed group did not use any type of personal protective equipment, as they reported. Of all the exposed groups who were working in garage sites, only 14 (38.9%) were not aware of lead exposure, while 22 (61.1%) of the exposed groups were aware of lead exposure. There were 14 (38.9%) participants in the exposed group who had received safety training. Among the exposed individuals in this study who used exposure prevention methods such as washing their hands before eating and taking showers after the completion of work, 21 (58.3%) were exposed. In terms of addiction habits (drinking alcohol), 15 (41.7%) of the exposed groups were dependent on consuming alcohol during work and after-work activities. According to the overall reporting of the study participants, 24 (66.7%) of the exposed groups did not follow occupational health and safety rules and regulations (safety practices) in the workplace. Of all participants in this study, approximately 31 (86.1%) of the exposed groups had a previous (prior) history of employment at other garage sites (Table 3).

**Table 3:** Behavioral characteristics of exposed groups (*n* = 36) in Addis Ababa, Ethiopia, 2023

Variables	Numbers of exposed workers	Percent (% )
PPE reporting		
Yes	19	52.8
No	17	47.2
Lead awareness		
Yes	22	61.1
No	14	38.9
Training on OSH		
Yes	14	38.9
No	22	61.1
Exposure prevention method		
Yes	21	58.3
No	15	41.7
Addiction to alcohol		
Yes	15	41.7
No	21	58.3
Knowledge of lead hazard		
Yes	10	27.8
No	26	72.2
Safety practice in the workplace		
Yes	12	33.3
No	24	66.7
Previous employment in a garage		
Yes	31	86.1
No	5	13.9

### Occupational job positions of the study participants

The higher and lower mean blood lead levels per occupational job position of the exposed groups were 35.5 ± 8.3 µg/dl and 24.1 ± 10.7 µg/dl, respectively. However, according to one-way ANOVA, there were no statistically significant differences (*p* > 0.05) in the mean blood lead levels per occupational job position among the mechanics, electricians, welders, or painters among the study participants (Table 4).

**Table 4:** Job position of exposed groups (*n* = 36) and unexposed groups (*n* = 34) in Addis Ababa, Ethiopia, 2023

Group	Occupational job position	Mean ± SD µg/dl	ANOVA, *p* value
Unexposed group	Health workers	15.3 ± 10	0.7
	Supportive staffs	13.9 ± 10.1	
Exposed group	Mechanics	30.6 ± 12.1	0.33
	Electricians	32.6 ± 15.2	
	Welders	24.1 ± 10.7	
	Painters	35.5 ± 8.3	

### Comparison of blood lead levels among the study participants

According to the independent sample t test, there was a significant difference between the exposed and unexposed groups (t (68) = 5.6, *p* < 0.001), with the mean blood lead level in the exposed group (M = 29.7, SD = 12.2, median = 27.5, and range = 6–52.5) µg/dl being greater than that in the unexposed group (M = 14.8, SD = 9.9, median = 15, and range = BDL-32.5) µg/dl). The magnitude of the two groups’ mean difference (mean difference = 14.9, 95% CI: 9.6 to 20.2) was statistically significant (Table 5).

**Table 5:** Comparison of blood lead levels between the exposed group (*n* = 36) and unexposed group (*n* = 34) in Addis Ababa, Ethiopia, 2023. (* significant at *p* < 0.05)

Group	Mean ± SD µg/dl	95% CI (µg/dl)	t	*P* value
Exposed group	29.7 ± 12.2	11.4–18.3	5.6	< 0.001*
Unexposed group	14.8 ± 9.9	25.6–33.8		

According to the one-way ANOVA, there were highly significant differences in the normal, acceptable, and dangerous blood lead levels among the exposed groups (*p* < 0.001). The mean blood lead levels of the exposed groups in the normal (5.6%) and dangerous (22.2%) BLL category ranges were 6.8 ± 1.1 and 46.9 ± 3.9 µg/dl, respectively, whereas in the normal (332.4%) and acceptable (67.6%) ranges of BLL, the mean blood lead levels of the unexposed groups were 3.3 ± 3 and 20.3 ± 6.7 µg/dl, respectively, with a highly significant difference (*P* < 0.001) between the normal and acceptable ranges of the unexposed groups (Table 6).

**Table 6:** Categorization of the BLLs of the study workers according to the OSHA recommendation limits. (* significant at *p* < 0.05)

BLL categories	Exposed groups (*n* = 36)			Unexposed groups (*n* = 34)		
	n (%)	Mean ± SD µg/dl	*P* value	n (%)	Mean ± SD µg/dl	*P* value
< 10 (normal)	2 (5.6)	6.8 ± 1.1	0.001*	11 (32.4)	3.3 ± 3	0.001*
10–40 (acceptable)	26 (72.2)	26.2 ± 7.3	0.001*	23 (67.6)	20.3 ± 6.7	
> 40 (dangerous)	8 (22.2)	46.9 ± 3.9	0.001*	-	-	

### Factors associated with the blood lead level of the study participants

According to multiple linear regression analysis, the combined significant effects on the blood lead level of the exposed groups according to the general model summary result were R^2^ = 0.856, adjusted R^2^ = 0.798, F [10, 25] = 14.87, *p* < 0.001. However, some predictors, such as extra working hours, service years, and previous (prior) employment in a garage, had a statistically significant effect on the increase in lead exposure. The mean blood lead levels of the exposed groups during extra working hours, service years, and previous (prior) employment in a garage increased by 3.8 µg/dl, 0.8 µg/dl, and 7.6 µg/dl, respectively, but other predictors had no direct impact on lead exposure (Table 7).

**Table 7:** Linear regression for exposed groups (*n* = 36) in Addis Ababa, Ethiopia, 2023

Variables	Coefficient (β)	S. E	LB at 95% CI	UB at 95% CI
Constant	-27.67	12.87	-54.18	-1.15
Age in years	0.31	0.25	-0.21	0.82
Extra working hours	3.8*	1.38	0.96	6.63
Long service years	0.8*	0.33	0.11	1.48
PPE reported (yes)	-4.17	2.9	-10.15	1.81
Lead awareness (yes)	-0.47	2.96	-6.58	5.63
Exposure prevention methods (yes)	-0.57	2.99	-6.74	5.59
Knowledge of lead hazards (yes)	-4.26	2.87	-10.17	1.66
Training on OSH (yes)	2.67	3.021	-3.56	8.89
Safety practice (yes)	0.04	2.876	-5.88	5.96
Prior employment in a garage (yes)	7.6*	3.129	1.01	13.89

LB: Lower Bound; UB: Upper Bound; * significant at *p* < 0.05; S.E.: standard error

## Discussion

A few studies on occupational exposure to blood lead levels among automotive garage workers were carried out previously in Jimma and Harar, Ethiopia [5, 12]. However, the blood lead levels of Anbessa City Bus Service Enterprise garage workers in Addis Ababa, Ethiopia, have not been studied previously. Hence, the results obtained in this study showed that the mean blood lead level (29.7 ± 12.2) 𝜇g/dl in the exposed group was significantly greater than the mean blood lead level (14.8 ± 9.9) 𝜇g/dl in the unexposed group (*p* < 0.001). These findings were similar to those of studies conducted in Pakistan and Nearby Addis Ababa-Adama Highway; however, these results were greater than those of studies conducted in Jimma, Ethiopia, for both exposed and unexposed groups [1, 5, 21]. The possible difference in this study could be due to differences in sample size, additional working hours, workload, lack of occupational safety training, and the nature of the working environment.

The World Health Organization (WHO) recommended that the health-based maximum individual biological action levels of male and female workers be 40 µg/dl and 30 µg/dl, respectively [22]. However, this study revealed that 22.2% of the exposed groups had blood lead levels that exceeded the maximum recommended blood lead level limit by the WHO, but this percentage did not exceed that of the unexposed group [22]. The World Health Organization (WHO) and Centers for Disease Control and Prevention (CDC) also recommended that the blood lead level standard limit of the general population be 10 µg/dl [22–24]. However, when we compared the blood lead levels of the study participants from the general population, 34 (94.4%) of the exposed and 23 (67.6%) of the unexposed groups of BLLs were above the general population’s BLL recommendation limit [22, 23]. The Occupational Safety and Health Administration (OSHA) and other agencies categorized the blood lead levels of workers into three stages: <10 µg/dl, 10–40 µg/dl, and > 40 µg/dl were considered normal, acceptable, and dangerous, respectively [25, 26]. However, when we compared both exposed and unexposed groups to OSHA and other agencies, 26 (72.2%) and 2 (5.1%) exposed and 23 (67.6%) and 13 (17.8%) unexposed groups were found to be in acceptable and normal ranges, respectively, but the maximum recommended blood lead level limit was 40 µg/dl [22, 27]. Compared with the National Institute for Occupational Safety and Health (NIOSH) standards, blood lead levels exceeded the recommended standard blood lead level limit of 5 µg/dl for approximately 57 (81.4%) of all study participants [28, 29]. However, the average blood lead levels (29.7 ± 12.2 µg/dl) of the exposed group and (14.8 ± 9.9 µg/dl) of the unexposed group were less than those of the OSHA (40 µg/dl), the European Union Scientific Committee on Occupational Exposure (30 µg/dl), the American Conference of Governmental Industrial Hygienists (30 µg/dl), and the WHO (40 and 30 µg/dl) recommended blood lead level limits for males and females, respectively [22, 30]. This indicates that in Ethiopia, there are no well-known blood lead level standard limits for occupational workers since Ethiopia follows the WHO and International Labour Organization (ILO) occupational and safety guidelines. In our study, statistically significant differences were not found between the occupational job positions of the exposed groups. A similar study conducted in Pakistan and Jimma, Ethiopia, did not find a statistically significant difference in occupational job positions [1, 31]. A possible explanation is that most automotive workers who were engaged in different occupational job positions in garages were affected by homogeneous lead exposure levels.

The main predictors of blood lead levels were analysed using multiple linear regressions. However, extra working hours, service years, and previous (prior) employment in a garage were the main statistically significant predictors and had 79.8% combined significant effects on the blood lead level of the exposed groups. A similar study reported that in Nigeria, the operation time was associated with a significant increase in blood lead levels according to automobile mechanics [9]. Similarly, studies conducted in Jimma, Ethiopia, and Iran have shown that exposure to blood lead has a direct relationship with additional working hours [2, 31]. This could be explained by the fact that extra working hours and a long duration of service were important factors in occupational lead exposure. As a result, workers who have been working in garages for long periods/years might be affected by acute and chronic health risks. This indicated that extra working hours and service years of exposed groups positively increased the exposure of blood lead levels of workers. In a similar study conducted in Nigeria, Harar, and Jimma, Ethiopia, long service years for automotive workers had adverse health effects on lead exposure [5, 9, 12]. However, in other studies that were conducted on the Addis Ababa-Adama Highway and Iran, service years had no direct significant effect on blood lead levels [21, 32, 33]. This difference might be due to the presence of lead exposures that can come from different environmental media (air, water, and soil) and the duration of work hours. Thus, non-garage workers and garage workers may be exposed to lead through the air they breathe, the water they drink, the foods they eat, and the surface materials they contact. The other significant variable that affected the blood lead level was a previous (prior) history of employment at other garage sites. Several studies have shown that garage workers are usually more affected by lead exposure than non-garage workers are [5, 12, 17, 34]. The production and recycling of lead-acid batteries, paints, soldering, and electronic wastes were greater at the garage sites than at the non-growth sites. As a result, most garage workers in devolving counties are currently exposed to lead and have experienced short- and long-term adverse health effects.

## Conclusion

In this study, there was a significantly greater mean blood lead level in the garage workers than in the non-garage workers. Blood lead levels exceeded the blood lead limits recommended by the WHO and OSHA (40 µg/dl) for approximately 22.2% of the exposed groups. Extra working hours (more than 8 h per day), a long duration of service and prior employment at garage sites were found to be the main significant predictors of elevated blood lead levels among garage workers. Thus, workers who are working in garage sites require more attention to safety training and preventive mechanisms to reduce exposure to lead and improve the hygiene practices of workers in each working department. Finally, regular checkups and longitudinal studies at the national level with large sample sizes are highly recommended.

## Data Availability

All the data analysed during this study are included in this article. The data that support the findings of this study are also available from the corresponding author, and the data are available at any time to the journal when a reasonable request is needed.
